# Long-Term Results of Adjunct Autologous Platelet-Rich Plasma in Lamellar Macular Hole Surgery Showing Lasting Restoration of Foveal Anatomy

**DOI:** 10.3390/ijms24054589

**Published:** 2023-02-27

**Authors:** Felix Hagenau, Elisa V. Osterode, Julian E. Klaas, Denise Vogt, Leonie F. Keidel, Benedikt Schworm, Jakob Siedlecki, Wolfgang J. Mayer, Thomas C. Kreutzer, Siegfried G. Priglinger

**Affiliations:** 1 Department of Ophthalmology, University Hospital, LMU Munich, 80336 Munich, Germany; 2 Department of Ophthalmology, University Medical Center Hamburg-Eppendorf, 20251 Hamburg, Germany

**Keywords:** lamellar macular hole, LMH, platelet-rich plasma, PRP, vitrectomy, peeling, Mueller cells, retina

## Abstract

The aim of this study was to evaluate the long-time results of highly concentrated autologous platelet-rich plasma (PRP) used as an adjunct in lamellar macular hole (LMH) surgery. Nineteen eyes of nineteen patients with progressive LMH were enrolled in this interventional case series, on which 23/25-gauge pars plana vitrectomy was performed and 0.1 mL of highly concentrated autologous platelet-rich plasma was applied under air tamponade. Posterior vitreous detachment was induced, and the peeling of tractive epiretinal membranes, whenever present, was performed. In cases of phakic lens status, combined surgery was carried out. Postoperatively, all patients were instructed to remain in a supine position for the first two postoperative hours. Best-corrected visual acuity (BCVA) testing, microperimetry, and spectral domain optical coherence tomography (SD-OCT) were carried out preoperatively and at minimum 6 months (in median 12 months) postoperatively. Foveal configuration was postoperatively restored in 19 of 19 patients. Two patients who had not undergone ILM peeling showed a recurring defect at 6-month follow-up. Best-corrected visual acuity improved significantly from 0.29 ± 0.08 to 0.14 ± 0.13 logMAR (*p* = 0.028, Wilcoxon signed-rank test). Microperimetry remained unchanged (23.38 ± 2.53 preoperatively; 23.0 ± 2.49 dB postoperatively; *p* = 0.67). No patients experienced vision loss after surgery, and no significant intra- or postoperative complications were observed. Using PRP as an adjunct in macular hole surgery significantly improves morphological and functional outcomes. Additionally, it might be an effective prophylaxis to further progression and also the formation of a secondary full-thickness macular hole. The results of this study might contribute to a paradigm shift in macular hole surgery towards early intervention.

## 1. Introduction

Any macular lesion can impair visual acuity, lead to metamorphopsia, and further result in reduced vision and quality of life. This also applies to lamellar macular holes (LMH), which were first described in 1976 by Gass et al., using slit lamp biomicroscopy, as oval reddish macular lesions without subjective hole formation in the gap of light presented to the patient [1].

Over time and with the introduction of high-resolution spectral domain optical coherence tomography (SD-OCT), partial-thickness macular defects were redefined by Hubschman et al. and the international vitreomacular traction study group and subdivided into LMH, ERM foveoschisis (ERM-FS), and pseudoholes. The diagnosis of LMH is based on the following OCT criteria: (1) irregular foveal contour, (2) foveal cavity with undermined edges, and (3) signs of tissue loss. In many cases, additional OCT signs may be present, such as epiretinal proliferation (EP), a foveal bump, and ellipsoid zone disruption [2,3].

To avoid misunderstandings, we use the terminus ERM-FS as synonymous to tractional lamellar macular holes (TLMH), which was proposed by Govetto et al., as the pathogenesis of both is identical [4,5]. ERM-FS appears as a “moustache”-like lesion with a sharp-edged split between the outer plexiform layer (OPL) and the outer nuclear layer (ONL) premacular membrane and an intact ellipsoid zone. In contrast, the LMH presents as “top hat”-shaped with round-edged cavitations, foveal bumps, epiretinal proliferation (EP; syn. to lamellar macular hole associated proliferation (LHEP)) and ellipsoid zone defects. The latter was first described by Pang et al. and manifests as a midreflective layer in the OCT [6,7]. EP seems to be composed mostly of proliferating and/or hypertrophied Mueller cells of the foveal walls that were disrupted and have migrated to the retinal surface [8]. In LMH without degenerative cavitations, EP is connected to the Mueller cell conus of the foveola. This tissue of medium reflectivity covers the whole inner surface of the LMH (non-elevated foveal walls) and connects the cell conus of the foveola with EP at the vitreous surface of the walls [5].

The current OCT classification distinguishes between the subentities that seem to be relevant in clinical routine with regard to progression [9]. The implications for morphological and functional outcomes after surgery are controversial and still being debated [10,11,12,13,14].

Whereas diagnostic criteria for LMH are precisely defined, there still exists no clear guideline for standardized treatment. Similarly, the benefits and especially the optimal timing of surgical intervention are still matters being resolved through discussion. While some studies only cautiously recommend surgical intervention, others show promising results with regard to visual and morphological outcomes [14,15,16,17].

Platelet-rich plasma (PRP) was first used in the 1990s for macular hole surgery. Promising results have been documented especially regarding its use for treating refractory, traumatic, or full-thickness macular holes [18,19,20]. Platelets are a natural reservoir of growth factors, e.g., epidermal growth factor (EGF), vascular endothelial growth factor (VEGF), and platelet-derived growth factor (PDGF) [21]. These are secreted when platelets come into contact with disintegrated tissue, such as after ILM peeling of lamellar macular holes, and therefore play a pivotal role in the regeneration of macular defects [22].

This has led to the use of platelets as an adjuvant in macular hole surgery to modulate wound healing processes and tissue remodeling, thus improving anatomical and visual outcomes. To date, only few clinical data are available for LMH surgery, especially for modifications such as highly concentrated autologous platelet-rich plasma [23,24], which was used in our study.

The aim of our study is to add to the knowledge of morphological and functional outcomes of lamellar macular holes undergoing vitrectomy with ILM peeling in combination with PRP.

## 2. Results

In total, 19 eyes from 19 patients with a symptomatic and progressive degenerative lamellar macular hole were enrolled in this interventional case study (Table 1). All patients fulfilled the SD-OCT-based main diagnostic criteria of degenerative lamellar macular holes.

The mean age at surgical intervention was 70 ± 9 years (median 71 years, range 56–82 years). There were 7 women (37%) and 12 men (63%). The mean follow-up was 14.2 ± 6.7 months (median: 12 months; range: 6–34 months).

Lens status was evenly distributed, with 8 pseudophakic and 11 phakic patients. All phakic patients underwent combined phacovitrectomy with phacoemulsification and implantation of an intraocular lens.

### 2.1. Morphological Findings

Preoperatively, all patients fulfilled the mandatory criteria for LMH on SD-OCT. Next to the irregular foveal contour, the foveal cavity with undermined edges and signs of foveal tissue loss, and associated alterations on SD-OCT, were present at the vitreoretinal interface, as shown in Table 1. Hyperreflective epiretinal tissue was used as an umbrella term to refer to (tractional) epiretinal membranes as well as the vitreous cortex, which are very challenging to distinguish using only SD-OCT.

**Figure 1:**
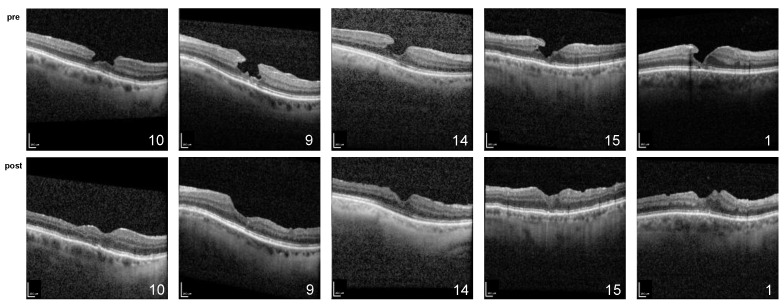
Spectral domain optical coherence tomography of patients (ID 1, 3, 7, 9, and 10) conducted preoperatively (**top**) and at the most recent follow-up (**bottom**); scale bar = 200 µm.

**Figure 2:**
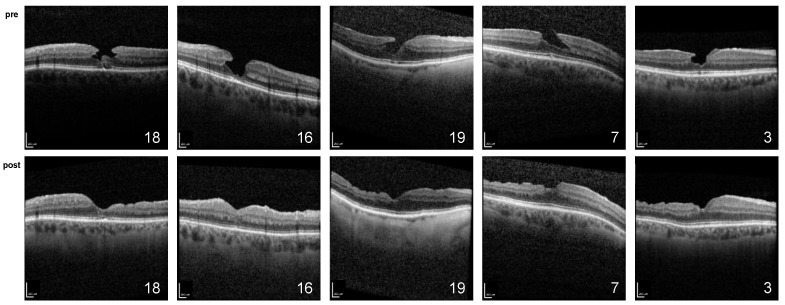
Spectral domain optical coherence tomography of patients (ID 14, 15, 16, 18, and 19) conducted preoperatively (**top**) and at the most recent follow-up (**bottom**); scale bar = 200 µm.

Initially, the restoration of the foveal contour with no signs of tissue loss remaining was observed in all cases (Figure 1 and Figure 2). Ellipsoid zone defects improved in 6 of 11 cases (55%). This morphology was stable during the whole follow-up period, except for in the cases of three patients, which are described below.

In two of three patients, there was a recurrent foveal defect present postoperatively at 6 months. Those patients were the only two of all the patients who had not received ILM peeling. The foveal defect was stable, with no functional decline over a follow-up of 12 months. Due to these findings of stability, a re-vitrectomy has not yet been performed.

The third patient disregarded recommendations to postoperatively remain in a supine position, which presumably led to PRP dislocation. A secondary vitrectomy with a reapplication of PRP was performed after the resorption of endotamponade. After 3 months, the foveal morphology was restored, and the functional parameters indicated improvement.

Postoperative cystoid macular edema was seen in 4 of 19 eyes (21%). These were treated with nonsteroidal anti-inflammatory eye drops (0.3% Nepafenac) and/or the parabulbar injection of 40 mg triamcinolon. The resolution of the macular edema was achieved in all cases.

The central macular thickness (CMT) in the 1 mm circle of an overlying ETDRS-grid changed from 304 ± 36 µm (median 299 µm range 240–378 µm) preoperatively to 314 ± 34 µm (median 314 µm range 244–392 µm) at the final follow-up.

### 2.2. Functional Outcomes

In all cases, best-corrected visual acuity (BCVA) showed statistically significantly improvements, comparing preoperative values of 0.33 ± 0.15 logMAR (median 0.30 logMAR, range 0.70–0.20 logMAR) with 0.18 ± 0.13 logMAR (median 0.10 logMAR, range 0.40–0.00 logMAR) at the last documented follow-up (*p* = 0.001, Wilcoxon signed-rank test).

In detail, subgroup analysis of pseudophakic patients only showed an improvement of BCVA from 0.34 ± 0.11 logMAR (median 0.35 logMAR, range 0.50–0.20 logMAR) preoperatively to 0.21 ± 0.12 logMAR (median 0.20 logMAR; range 0.40–0.10 logMAR) at the last documented follow-up, which was also found to be statistically significant (*p* = 0.047, Wilcoxon signed-rank test). Microperimetry ranged from a preoperative mean threshold of 23.39 ± 2.43 dB (median 23.80 dB, range 18.50–26.80 dB) to a mean threshold of 22.26 dB (median 23.50 logMAR, range 14.40–25.90 logMAR; not statistically significant, *p* = 0.51) at the end of the follow-up period.

Fixation stability P1 (preoperatively 72.35 ± 29.00%, end of follow-up 72.50 ± 29.07%; *p* = 0.422) and P2 (preoperatively 90.18 ± 13.70%, end of follow-up 89.81 ± 16.27%, *p* = 0.527) did not indicate any statistically significant changes. Subjective metamorphopsia was postoperatively reduced in all cases.

## 3. Discussion

In this interventional case study of 19 patients with progressive LMH treated by pars plana vitrectomy with ILM peeling and highly concentrated autologous platelet-rich plasma, we could observe morphological and functional improvement at the long-term follow-up. The prevention of further progression even into stages that are more visually limiting thus also seems to argue for earlier surgical intervention.

Until now, no national nor international guidelines have been established for the management of partial-thickness macular holes. Therefore, whether the correct approach is to treat or to not treat lamellar macular holes is still a matter of discussion.

A very important step toward shedding light on this question is the new classification of Hubschman et al., which allows differentiating between the distinct entities of partial defects [2]. Thus, the results of previous studies have to be taken with caution, as the terminology has not been clearly defined, and partial-thickness defects must also be individually addressed.

Considering the results of our research, two different results have to be taken into account. On the one hand, there was morphological improvement of the foveal structure and prevention of progression. On the other hand, the measurements show that visual acuity is functionally improved.

### 3.1. Morphological Improvement

The pathogenesis of LMH is not yet been fully understood, nor are the exact mechanisms of regeneration after macular surgery known, especially in combination with PRP. While, in most cases, degenerative LMH seems to remain stable or may even close spontaneously over time [25], in others, a progressive degenerative natural course is observed, with the development of ellipsoid zone defects and possible conversion into a full-thickness macular hole (FTMH) [26]. Two mechanisms seem to be important for foveal restoration after vitreomacular surgery:

(1) Release of the vitreous adhesion/traction: Peeling of the vitreous cortex together with ILM results in the closure of the cavitations, but retinal layers still appear disrupted [9]. Microstructure continues to be disorganized, and cavitations are replaced by midreflective material. Improving structural defects and halting the degenerative process thus seems to require additional surgical modifications in form of, e.g., PRP.
(2) Activation of Mueller cells and stabilization of the foveal microenvironment: Mueller cells account for 90% of the retinal glia and play a pivotal role in retinal wound healing [27]. ILM peeling leads to the shaving of the basal membrane of Mueller cells, which acts as a stimulus for proliferation [27]. PRP acts as an important factor in further supporting the healing process. PRP is composed of platelets that are activated through contact with disintegrated neuroretinal tissue. They are known to be rich in growth factors and cytokines such as vascular endothelial growth factor (VEGF), platelet-derived growth factor (PDGF), epidermal growth (EGF), fibroblast growth factor (FGF), insulin-like growth factor 1 and 2 (IGF-1, IGF-2), transforming growth factor beta 1 (TGFβ1), and cytokines [21]. Of these, PDGF, EGF, IGF-1, and FGF seem to be the most relevant [22]. Thereupon, signal transduction pathways are activated in Mueller cells and regulate migration, proliferation, and tissue remodeling [27].

As we did not observe any secondary FTMH in our study, such an optimized microenvironment may well preclude the development of FTMH. PRP seems to additionally improve the success rate of complete defect closure, increasing the likelihood of superior foveal architectural restoration and, consequently, functional improvement.

Regarding defects in the ellipsoid zone, which are a sign of chronicity, we observed restoration after vitrectomy with PRP over time in 6 of 11 cases (54.5%). The status of the foveal external limiting membrane (ELM) and the ellipsoid zone (EZ) is correlated with central retinal sensitivity and BCVA. Therefore, some authors have proposed that restoration of foveal configuration is not the only important factor for BCVA improvement but, rather, that continuity of the ellipsoid zone seems to be more essential [28,29]. The number of patients in our study is unfortunately too low to allow the evaluation of these statements on a statistically convincing basis. The results are, however, consistent with the observations of Holland et al., who described improved preoperative to postoperative visual acuity due to fewer ellipsoid zone defects. Based on this finding, one should consider earlier surgical intervention in LMH patients before the development of ellipsoid zone defects [30].

### 3.2. Functional Improvement

One of the main reasons why vitrectomy in partial-thickness macular holes is still controversial is the reduced functional benefit found in a few prior studies [10,11].

In accordance with most of the available publications, this study demonstrates a significant postoperative increase in the visual acuity of phakic (0.15 logMAR) and pseudophakic (0.13 logMAR) patients [12,14,31]. A recent metanalysis of Parisi et al. reported the surgical outcomes for 463 eyes with tractive or degenerative LMH from 13 studies [32]. In these studies, the increase in visual acuity after surgical intervention ranged from 0.1 to 0.21 logMAR. Taking a closer look at studies with the largest improvement in visual acuity, such as Obata et al. with an increase of 0.21 logMAR, it must be considered that functional improvement may only be due to cataract surgery, as, of the 13 included patients, 12 received combined phacovitrectomy [33]. Coassin et al. studied 106 symptomatic LMH patients that either underwent a simple PPV or phacovitrectomy and experience significant improvements in postoperative BCVA (*p* < 0.001) [34]. When phacovitrectomized patients were excluded from the analysis, there was still significant improvements in postoperative BCVA (*p* = 0.0036), as was the case with the pseudophakic subgroup in our study.

The subgroup analysis of pseudophakic patients or—even better—prospective trials with a homogenous, pseudophakic cohort will be very important to eliminate this confounder.

In terms of safety aspects, the use of PRP as an adjunct therapy did not cause any additional complications and, in particular, did not lead to the loss of visual function. However, there are two factors that have to be considered when using PRP. The widely discussed ILM peeling seems to be mandatory because it was not performed in the two cases in which we saw a recurrent defect. This has led to the hypothesis that PRP needs to come into direct contact with disintegrated tissue to be activated [22,27]. The second important factor is the postoperative supine positioning of the patient for 1–2 h. Ignoring this might lead to PRP dislocation. The different endotamponades do not seem to have a significant influence on the results; however, air tamponade seems to be sufficient and the preferable choice due to the short resorption time.

Another promising method is the EP embedding technique, where the EP material is placed in the foveal defect [35]. Considering the hypothesis that EP is formed as an attempt by Mueller cells to regenerate the foveal tissue defect, there are similarities here to the hypothesized mode of action of PRP. The common factor is the activation of the Mueller cells.

While our results, but also those of other groups, demonstrate a reason for surgical intervention, the exact surgical procedure with any necessary modifications or use of adjuvants is still a matter of discussion. One possibility is the use of highly concentrated autologous platelet-rich plasma. To date, our study has the largest cohort with the longest period to follow-up of degenerative lamellar macular holes undergoing vitrectomy with peeling and the use of PRP. The additional use of PRP as an adjuvant might further enhance the morphological and functional outcomes and, even more importantly, is able to prevent the progression of LMH to stages of high vision impairment. Therefore, early surgical intervention seems reasonable.

Our study is limited by its small sample size, lack of control group, and inhomogeneous lens status. Further studies are needed to compare the advantages of the different techniques and approaches and to determine the most efficient method. Authors should discuss the results and how they can be interpreted from the perspective of previous studies and of the working hypotheses. The findings and their implications should be discussed in the broadest possible context. Future research directions may also be highlighted.

## 4. Materials and Methods

### 4.1. Study Design

We included 19 eyes from 19 patients with progressive and symptomatic lamellar macular holes in this prospective, interventional case series. All eyes underwent 23-gauge vitrectomy in combination with an endotamponade (SF6, C2F6) and with the application of autologous, highly concentrated platelet-rich plasma. Surgery was performed by highly experienced vitreoretinal surgeons (SGP, TCK, and WJM) at the Department of Ophthalmology, Ludwig Maximilian University of Munich, Germany. Surgery was carried out between December 2019 and November 2022.

The study was approved by the institutional review board of the University Eye Hospital of the Ludwig Maximilian University of Munich and was conducted in accordance with the tenets outlined in the Declaration of Helsinki. All subjects provided written informed consent before undergoing the interventions as described below. The literature research was carried out via PubMed^®^ of the National Library of Medicine, and relevant scientific publications were selected.

### 4.2. Patient Selection

Clinical examination and multimodal imaging, including SD-OCT, were performed on all patients. SD-OCT-based diagnostic criteria of LMH were met when the fovea showed (1) an irregular contour, (2) undermined edges, and (3) signs of tissue loss [2]. Patients with concomitant retinal pathologies such as diabetic retinopathy, vitreous hemorrhage, retinal detachment, age-related macular degeneration, inflammatory disease, vascular occlusion, high myopia ≥ −6.00 dpt, or trauma were excluded.

Surgery was recommended when at least two of the following findings occurred during the preoperative follow-up period: (1) significant reduction in visual acuity, (2) progression of the foveal morphology, and/or (3) significant impairment of quality of life caused by metamorphopsia.

All patients were evaluated preoperatively and at minimum 6 months or longer after surgery, which included identical work up. Potential postoperative complications, e.g., macular edema, were recorded at any time point during the follow-up period.

We determined best-corrected visual acuity (BCVA) using standard ETDRS charts at 4 m after subjective manifest refraction had been measured. The examination consisted of slit lamp biomicroscopy and included dilated fundus examination, SD-OCT scanning with volume and radial scans (SPECTRALIS HRA + OCT, Heidelberg Engineering,

Heidelberg, Germany), microperimetry (MAIA, Centervue Inc., Fremont, CA, USA), and fundus photography (Optos P200Tx, Optos, Dunfermline, UK).

### 4.3. PRP Preparation

PRP preparation was performed as described in previous publications [23,24]. Whole blood (105 mL) was drawn and anticoagulated at a ratio of 1:7. Separation into platelet-rich plasma, red blood cells, and platelet-deficient plasma was conducted using a special closed-circuit centrifugation method (Arthrex Angel System TM, Arthrex, Naples, FL, USA). Highly concentrated PRP is characterized by a low fraction of pro-inflammatory leucocytes and an 8.8× higher concentration of platelets than in whole blood.

### 4.4. Surgical Procedure

The procedure of 23-/25-gauge pars plana vitrectomy was performed by highly experienced surgeons through induction of posterior vitreous detachment and peeling of epiretinal tissue, if present, and ILM except in two patients, as described in Table 1.

After staining with MembraneBlue-Dual Dye (0.125 mg Brilliant Blue G and 0.75 mg Trypan Blue D.O.R.C., Zuidland, The Netherlands) peeling was conducted followed by a second control staining. All phakic patients underwent combined phacovitrectomy with implantation of a previously calculated intraocular lens. After gas (SF6, C2F6) or air tamponade, highly concentrated PRP (0.1 mL) was added to the posterior pole. Patients were strongly recommended to postoperatively remain in a supine position for 2 h.

### 4.5. Main Outcome Measures

Primary anatomical success was defined as hole closure and postoperative morphology on SD-OCT, such as integrity of the inner and outer retinal layers and the inner foveal contour during all follow-up scans. Secondary endpoints were functional results and included best-corrected visual acuity, microperimetry, and appraisal of metamorphopsia.

### 4.6. Statistical Analysis

Statistical analysis was performed using IBM SPSS Statistics Version 26 (IBM Corporation, New York, NY, USA). All data are presented as the means ± SD unless otherwise stated. The Wilcoxon signed-rank test was used to compare two related groups (BCVA, central retinal thickness, data of microperimetry). Values of *p* ≤ 0.05 were considered to indicate statistically significant differences.

## 5. Conclusions

This is an interventional case study with—to the best of our knowledge—the largest cohort of patients with progressive and symptomatic LMH undergoing vitrectomy with ILM peeling and the use of highly concentrated autologous platelet-rich plasma (PRP).

Using PRP as an adjunct was shown to improve morphological and functional outcomes, as well as to prevent further progression as assessed at long-term follow-up. For treatment of LMH, the use of PRP seems to be more effective than conventional surgery. Most importantly, the results show that the intervention can be seen as prophylaxis to secondary degenerative full-thickness macular hole formation and, thus, further vision loss. The data support early surgical intervention, which could lead to a paradigm shift in macular hole surgery.

## Figures and Tables

**Table 1:** Clinical and surgical data. M, male; F, female; OD, right eye; OS, left eye; IOL, intraocular lens; EP, epiretinal proliferation; HET, hyperreflective epiretinal tissue; ERM, epiretinal membrane; ILM, internal limiting membrane; VC, vitreous cortex; BCVA, best-corrected visual acuity; FU, follow-up.

ID	Age	Sex	Eye	Lens Status		VRI Findings	Surgery Peeling of Structures	BCVA [logMAR]		Follow Up Time
				pre-op	post-op			pre-op	Longest FU	[months]
1	78	M	OD	IOL	IOL	ERP, HET	ERM, ILM	0.4	0.1	34
2	81	M	OS	phakic	IOL	ERP	None	0.2	0	12
3	57	F	OD	phakic	IOL	ERP, HET	VC, ERM, ILM	0.2	0.2	9
4	67	F	OD	phakic	IOL	ERP	ERM, ILM	0.2	0	12
5	65	M	OS	IOL	IOL	ERP	ERM, ILM	0.4	0.3	24
6	71	M	OS	phakic	IOL	ERP, HET	VC	0.3	0.1	12
7	61	F	OD	phakic	IOL	HET	VC, ERM, ILM	0.2	0.1	12
8	80	M	OD	IOL	IOL	ERP, HET	VC, ILM	0.2	0.1	12
9	76	F	OS	IOL	IOL	ERP	ILM	0.3	0.3	26
10	71	M	OD	IOL	IOL	ERP, HET	ERM, ILM	0.2	0.3	12
11	78	F	OD	phakic	IOL	ERP	ILM	0.4	0.4	9
12	79	M	OD	IOL	IOL	ERP, HET	VC, ILM	0.4	0.1	12
13	56	M	OS	phakic	IOL	ERP, HET	VC, ILM	0.2	0.1	12
14	63	F	OD	phakic	IOL	ERP	ERM, ILM	0.6	0.4	17
15	69	M	OS	phakic	IOL	ERP, HET	ERM, ILM	0.2	0.1	13
16	56	F	OS	IOL	IOL	ERP	ILM	0.3	0.1	12
17	71	M	OD	phakic	IOL	ERP, HET	VC, ILM	0.7	0.2	6
18	82	M	OS	IOL	IOL	ERP, HET	ERM, ILM	0.5	0.4	11
19	75	M	OD	phakic	IOL	ERP, HET	ERM, ILM	0.4	0.2	13

## Data Availability

All data and analysis can be sent upon request.
